# Special Issue: ‘Pathogenesis of Emerging Zoonotic Viral Infections’

**DOI:** 10.3390/pathogens11070736

**Published:** 2022-06-29

**Authors:** Toshana L. Foster, Svetlana F. Khaiboullina

**Affiliations:** 1 Faculty of Medicine and Health Sciences, School of Veterinary Medicine and Science, The University of Nottingham, Sutton Bonington, Loughborough LE12 5RD, UK; toshana.foster@nottingham.ac.uk; 2 Institute of Fundamental Medicine and Biology, Kazan Federal University, 420008 Kazan, Russia; 3 School of Medicine, University of Nevada, Reno, NV 89557, USA

Emerging zoonotic infections present a serious global health threat [1]. It is estimated that approximately 60% of known infectious human diseases are caused by zoonotic transmission from domestic and wild animals, and an alarming 75% of all emerging infectious diseases are zoonotic [2]. Over the past two decades, several novel emerging viruses have caused local outbreaks, epidemics and pandemics characterized by high death rates and high incidences of post-infection impairment. The severe acute respiratory syndrome coronavirus 2 (SARS-CoV-2) pandemic has revealed the urgent need for early identification of potential threats and thus the timely development of measures for treatment and prevention [3].

It is reasonable to anticipate that the number of outbreaks and pandemics could increase in the near future. This assumption is based on multiple factors such as increased frequency of travel, particularly to remote areas, climate change, intensified global trade, susceptibility to pathogen exposure and several more [4]. In addition, virus spillover events and adaptations to novel reservoir host species due to virus genome mutations could advance pathogen spread within communities [5]. These factors, amongst others, significantly contribute to the frequency of human contact with reservoir hosts, thus increasing the risk of pathogen spillover to human populations. 

The SARS-CoV-2 pandemic has emphasized global weaknesses in the detection and control strategies aimed to minimize the human health and socioeconomic impact of exposure to zoonotic pathogens [6]. It is clear that there is an urgent need to better understand the nature of zoonotic pathogenesis [7]. Specifically, learning more about the mode of transmission and mechanisms of disease pathogenesis is the main priority of current research. Importantly, improvement of the diagnosis, treatment and prevention measures may only be possible when we have a deep understanding of the nature of pathogens, source and cause of disease emergence and mechanisms underlying infection and disease [8]. Further, analyzing the genetic diversity and the molecular mechanisms of host innate and adaptive immune responses to infection and learning more about host-pathogen co-evolution could help predict future outbreaks and identify novel targets for effective vaccines and therapeutics [9].

A major obstacle in our progress towards developing novel therapeutics and vaccines is the limited knowledge of the reservoir host disease dynamics, virus-recipient host interactions and the detailed processes that permit the establishment of infection [10]. This is explained by the diversity in virion structure, mode of infection, target cells, replication mechanisms of viruses and the physiological and immunological susceptibility of the recipient host, amongst other characteristics. The urgent need to understand the pathogenesis of emerging infections was demonstrated during the SARS-CoV-2 pandemic. It became apparent that early identification of the emerging pathogen is essential for the selection of pertinent approaches for exposure prevention and the development of treatment measures and prophylaxis [11]. Furthermore, previously accumulated knowledge of members of the same virus family and genera could prompt the implementation of the most effective and appropriate measures. 

In this Special Issue, we presented manuscripts (two experimental publications and two review articles) providing an insight on the distribution, spread, and protein functions of SARS-CoV-2, Puumala orthohantavirus and Ebola viruses. 

In the original research manuscript, Au and colleagues addressed the important issue of detecting and analyzing SARS-CoV-2 virus transcription in infected cells [12]. Although several studies have focused on studying the transcription of SARS-CoV-2 at specific timepoints, our understanding of the virus’s replication kinetics in infected cells requires further research efforts. In addition, the authors state the importance of the development of quick and highly specific systems that can detect variants of SARS-CoV-2 identified during the pandemic [12]. Understanding the mechanisms of viral transcription could help novel therapeutic developments aimed at restricting virus production. In this manuscript, the authors developed 28 ddPCR assays and defined specific primer and probe sets to analyze the RNA levels of 15 NSPs, 9 ORFs, and 4 structural genes. The transcriptional kinetics of these viral genes were followed for 12 h post-infection in Caco-2 cells. It was demonstrated that the initiation of viral transcription takes up to 6 h post-infection with SARS-CoV-2 [12]. These results contribute to a better understanding of SARS-CoV-2 infection mechanisms and provide insight into appropriate timeframes to administer antiviral therapy.

In another original study by Davidyuk et al., the genetic diversity of Puumala orthohantavirus (PUUV) in the Republic of Tatarstan (RT) was analyzed [13]. PUUV primarily causes a hemorrhagic fever with renal syndrome (HFRS) in the RT, which is one of the most active endemic regions in Russia. The goal of the study presented by the authors was to analyze genetic variations and determine the mechanisms behind these variations. The authors analyzed nucleotide sequences from a complete S segment and partial M and L segments from PUUV-positive bank voles. All PUUV strains were of the RUS genetic lineage and formed two subclades: the Western and Eastern Trans-Kama. PUUVs from Western Trans-Kama were similar to the Teteevo strain from the Pre-Kama clade [13]. These results suggest that the Teteevo Pre-Kama strain could have been introduced from Western Trans-Kama by the migration of bank voles. Therefore, it appears that, as a result of PUUV-infected bank vole migration, divergent PUUV strains could emerge in closely located populations, thus facilitating the genetic exchange between PUUV hosted within those populations [13].

This Special Issue also features review manuscripts on Ebola virus protein function, structure, and immune properties. The manuscript by Jain et al. presents a detailed analysis and discussion of the multi-functional features of each Ebola virus (EBOV) protein [14]. EBOV belongs to the Ebolavirus genus, family Filoviridae. The virus genome represents a negative-sense, non-segmented, single-stranded RNA that contains seven genes [14]. All genes encode for a single protein: nucleoprotein (NP), viral protein 35 (VP35), VP40, glycoprotein (GP), VP30, VP24, and RNA polymerase (L), except for the three pre-proteins that are released from the GP precursor [14]. The authors have highlighted multiple proteins and amino acid (aa) sites with potential targets for vaccine development; that is, by targeting NP at aa 110–400 and VP24 at aa 169–173, NP formation and viral replication were shown to be suppressed in infected cells. Virus replication could also be inhibited when blocking VP35 at position aa 210 and L at aa 741–744 and, concordantly, by binding to GP at aa 54–213, the release of the virus could be blocked. The evasion of the immune responses is essential for successful virus replication, and this can be accomplished by the expression of VP35, soluble secreted glycoprotein (sGP), shed GP, and VP24 proteins. To restrict virus interference with the immune response targeting specific regions of these proteins, such as VP30 aa 221–340 and VP24 aa 120–190, could be a potential therapeutic approach [14]. Thus, in this review, the authors provide a comprehensive summary of the differing and complementary roles of EBOV proteins in disease pathogenesis and their potential as therapeutic and vaccine targets.

In the final review, Jain et al. present a concise description of the cell types targeted by EBOV [15]. In addition, the authors discuss Ebola virus disease (EVD) symptoms and provide a comprehensive analysis of immune responses, both innate and adaptive. The authors summarize the regulation of immune responses by EBOV that contribute to the pathogenesis and severity of EVD. EVD is characterized by severe bleeding and multiorgan failure leading to patient death. In the last six years, three EBOV outbreaks have been documented and were characterized by a significant morbidity and mortality. This virus has multiple entry mechanisms, contributing to the high infectivity rates and rapid human-to-human transmission, and it exploits multiple immune evasion mechanisms, which warrants advances in effective vaccine and drug development as discussed in this review [15]. 

Collectively, this Special Issue highlights the proficiency with which viruses are able to cause severe disease and the complexities of virus–host interactions that impact both disease pathology and outcome. The knowledge attained from the articles covered here could aid the development of more specific peptide-based antivirals, monoclonal antibodies, and novel vaccines that protect against infections in the future. This area of research is absolutely essential and is urgently needed for better preparation for future pandemics.

We wish to express our appreciation to all the authors who have participated in this Special Issue and the reviewers for their comments.
